# *EEF1D*‐Associated Neurodevelopmental Disorders: Evidence From Iranian Siblings With a Biallelic Splice Junction Loss Variant

**DOI:** 10.1002/mgg3.70287

**Published:** 2026-10-21

**Authors:** Maedeh Ganji, Arastoo Kaki, Mohammad Farid Mohammadi, Javad Behroozi, Seyed Mohammad Kahani, Behzad Rezaei, Afshin Fayazi, Pouria Mohammadi, Ali H. Bereshneh, Masoud Garshasbi

**Affiliations:** ^1^ Department of Medical Genetics Isfahan University of Medical Sciences Isfahan Iran; ^2^ Department of Medical Genetics, School of Medicine Hamadan University of Medical Sciences Hamadan Iran; ^3^ Department of Cell and Molecular Sciences, Faculty of Biological Sciences Kharazmi University Tehran Iran; ^4^ Department of Medical Genetics, Faculty of Medical Sciences Tarbiat Modares University Tehran Iran; ^5^ Research Center for Cancer Screening and Epidemiology AJA University of Medical Sciences Tehran Iran; ^6^ Institute of Health Shiraz University of Medical Sciences Shiraz Iran; ^7^ Departments of Pediatrics, School of Medicine Hamadan University of Medical Sciences Hamadan Iran; ^8^ Myelin Disorders Clinic, Children's Medical Center, Pediatric Center of Excellence Tehran University of Medical Sciences Tehran Iran

**Keywords:** *EEF1D*, global developmental delay, microcephaly, neurodevelopmental disorders

## Abstract

**Background:**

Global developmental delay (GDD) and intellectual disability (ID) are neurodevelopmental conditions with significant phenotypic and genetic heterogeneity. *EEF1D* (OMIM* 130592) has recently been identified as a gene of interest in neurodevelopmental disorders. Neurodevelopmental disorder with thin corpus callosum, hypotonia, and absent language (NEDTCHAL) (OMIM #621150) is caused by homozygous or compound heterozygous disease‐causing variants in the *EEF1D*.

**Methods:**

We performed whole exome sequencing (WES) on an Iranian sibling presenting with global developmental delay, intellectual disability, microcephaly, and failure to thrive. Bioinformatic analysis and Sanger sequencing were used to validate and characterize the identified variant.

**Results:**

A novel homozygous splicing variant in *EEF1D* (c.1489‐26_1493del) was identified in both affected individuals. The variant is predicted to disrupt splicing and cause a frameshift deletion. Previous studies have implicated biallelic *EEF1D* variants in neurodevelopmental disorders, and this report is the first from the Iranian population and the seventh family to be reported worldwide with this rare condition.

**Conclusion:**

This report provides further evidence for the association between *EEF1D* variants and neurodevelopmental disorders, expanding the phenotypic spectrum of *EEF1D*‐related conditions. It reinforces the importance of recognizing *EEF1D* as a key gene involved in developmental delay and intellectual disability.

## Graphical abstract

We identified a novel homozygous variant (c.1489‐26_1493del) in *EEF1D* in an Iranian sibling with global developmental delay, intellectual disability, microcephaly, and failure to thrive. This likely pathogenic variant, absent in gnomAD v4, further supports *EEF1D* as a causative gene for autosomal recessive neurodevelopmental disorders, reinforcing findings from four previously reported families.

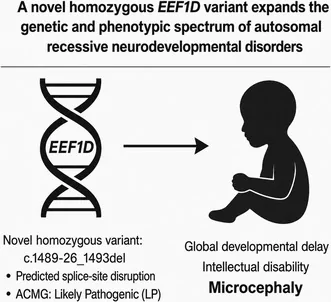

## 1 Introduction

Neurodevelopmental disorders encompass a wide range of conditions characterized by significant phenotypic and genetic heterogeneity. Recent studies have expanded the spectrum of *EEF1D*‐related neurodevelopmental disorders, identifying biallelic variants in *EEF1D* as a major contributor to these conditions (Najmabadi et al. 2011; Reuter et al. 2017). The phenotypic presentation associated with these variants includes intellectual disability, global developmental delay, microcephaly, and spasticity, as well as more specific neurological features such as a thin corpus callosum, hypotonia, and absent language. One such condition, Neurodevelopmental disorder with thin corpus callosum, hypotonia, and absent language (NEDTCHAL, OMIM # 621150), represents a severe form of these disorders, highlighting the critical role of *EEF1D* in brain development. This disorder further underscores the importance of understanding the genetic underpinnings of neurodevelopmental conditions and expanding our knowledge of *EEF1D* involvement in various phenotypic presentations (Najmabadi et al. 2011; Reuter et al. 2017; Averdunk et al. 2023; Zhang et al. 2024).

Disease‐causing variants in several genes encoding subunits of the translation elongation factor 1 (eEF1) have been linked to intellectual disability and neurodevelopmental disorders, which demonstrates the essential role of elongation stage of translation in neurodevelopment (McLachlan et al. 2019). The Eukaryotic translation Elongation Factor 1 Delta (*EEF1D*) (OMIM* 130592) is a protein coding gene which is responsible for the enzymatic delivery of aminoacyl tRNAs to the ribosome (Guo and Schimmel 2013; Kaitsuka and Matsushita 2015). This subunit functions as a guanine nucleotide exchange factor. This gene is versatile, producing at least four distinct protein isoforms in humans, as indicated by ENSEMBL Gene ID: ENSG00000104529. These isoforms are categorized into short and long types. The short isoforms, known as eEF1Bδ1, eEF1Bδ2, and eEF1Bδ3, comprise 281, 262, and 257 amino acids respectively. In contrast, the long isoform, eEF1BδL, contains 647 amino acids (Figure 1) (Kaitsuka and Matsushita 2015). The significant length disparity between these polypeptides is due to a large coding exon that is excluded from the short isoform transcripts but included in the long isoform transcripts (McLachlan et al. 2019). The standard eEF1Bδ isoform functions as a translational elongation factor within the cytoplasm, becoming part of the eEF1 complex. Meanwhile, the eEF1BδL isoform, containing a nuclear localization signal (NLS) at its N‐terminus, is transported to the nucleus where it serves as a heat shock response (HSR) transcription factor (Kaitsuka et al. 2011).

**FIGURE 1:**
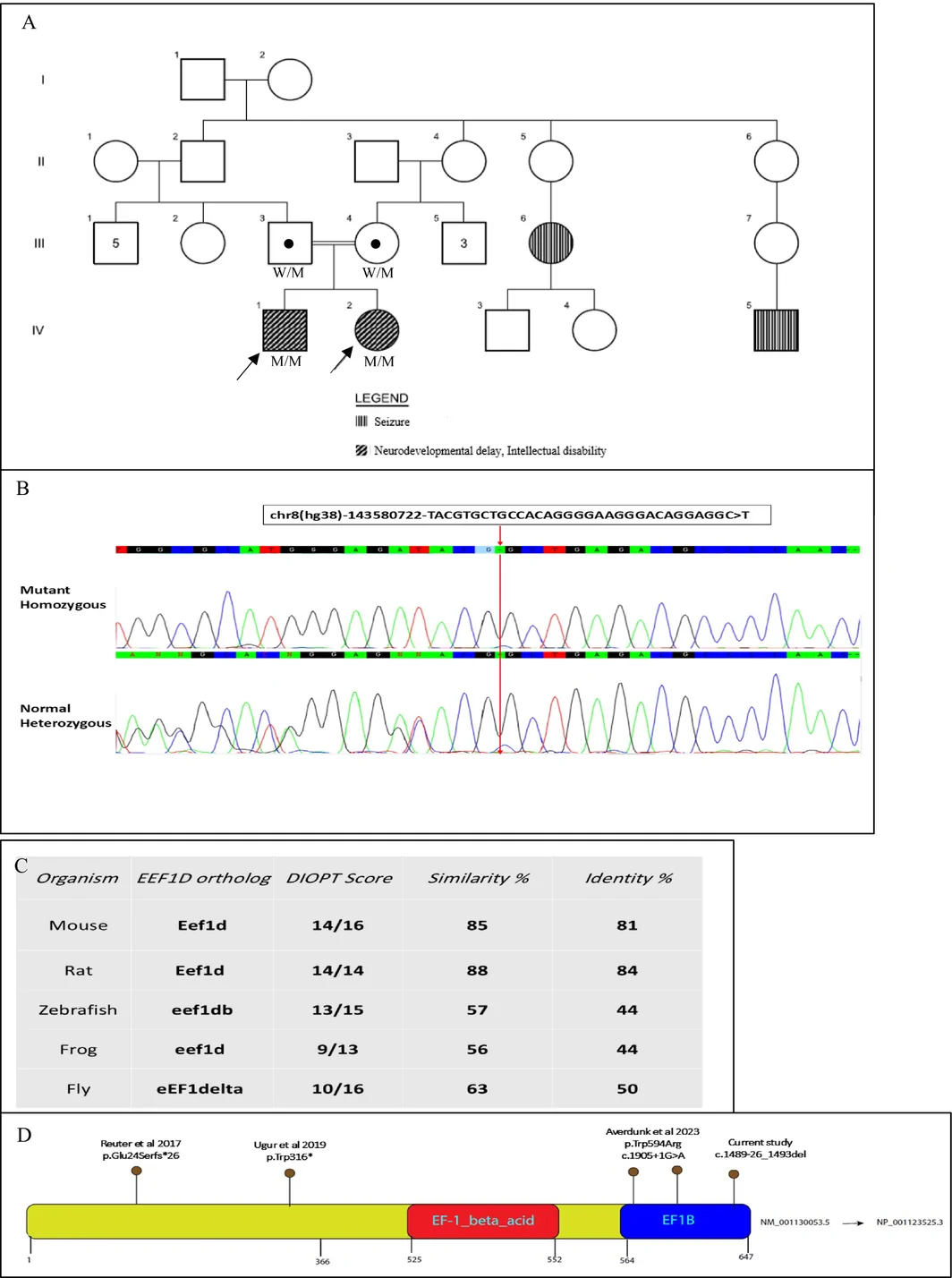
(A) Family pedigree of the patients. The affected probands are shown by arrow. The genotype of the parents for the variant is depicted on the pedigree as wild/mutant (W/M) and patients as mutant/mutant (M/M). (B) Sanger sequencing chromatographs shows heterozygous and homozygous status. (C) *EEF1D* remained conserved during evolution. (D) The schematic representation of the EEF1D protein (NP_001123525.3) highlights key mutations reported in various studies. The EF‐1 Beta Acid domain (red, residues 525–552) and the EF1B domain (blue, residues 564–647) are critical regions essential for proper protein function. Identified variants, including p.Glu24Serfs*26, p.Trp316*, p.Trp594Arg, c.1905+1G>A, and c.1489‐26_1493del, are mapped along the protein sequence. In the current study, the c.1489‐26_1493del variant affects the EF1B domain.

In 2017, for the first time, Reuter and colleagues identified a homozygous frameshift variant (p.Glu24Serfs*26) in *EEF1D* as a candidate gene for autosomal recessive intellectual disability in a Syrian family with three affected children, presented with intellectual disability, microcephaly, and short stature (Reuter et al. 2017). To the best of our knowledge, only six variants in *EEF1D* have been reported (Table 1). In the current study, we describe the first Iranian family with a homozygous splice junction loss variant in *EEF1D* in a sibling who presented with intellectual disability, seizure, neurodevelopmental delay, microcephaly, failure to thrive, cyanosis, and strabismus, with normal EEG and metabolic profile.

**TABLE 1:** Genetic and clinical findings of *EEF1D* in the literature review and present study.

	Reuter et al. (2017) (PMID: 28097321)			Ugur Iseri et al. (2019) (PMID: 30787422)			Averdunk et al. (2023) (PMID: 36576126)				Zhang et al. (2024) (PMID: 38083972)	Present study		Frequency (*n* = 13; 4 M, 9 F)
Gender	F	M	M	F	F	F	F	F	F	F	M	M	F	
Affected number	2 M, 1 F			3 F			2 F		2 F		1	1 M, 1 F		
Age	NA			12, 8, 4 years			5, 6 years		10, 22 years		3 years and 11 months			
Affected relation	Siblings			Siblings			Siblings		Double first cousins		−	Siblings		
Ethnicity	Syria			Turkey			Turkey		Oman		China	Iran		
Consanguinity	+			+			+		+		−	+		
EEF1D variant coordinate														
c.DNA	c.69del			c.948G>A			c.1780T>C		c.1905+1G>A		c.1905+1G>A	c.1489‐26_1493del		
											c.676C>T			
Exon	Exon 3/10			Exon 3/10			Exon 9/10		Intron 9/9		Intron 8/9	Intron 7/9		
											Exon 3/10			
Protein	p.(Glu24SerfsTer26)			p.(Trp316Ter)			p.(Trp594Arg)		−		−	−		
											p.(Gln226Ter)			
Effect	Frameshift			Nonsense			Missense		Splicing variant		Frameshift	Splice junction loss		
											Nonsense			
Zygosity	Homo			Homo			Homo		Homo	Homo	Compound het	Homo		
Neurological features														
Global developmental delay	+			+			+				+	+		13/13
Intellectual developmental delay	+			+			+				+	+		13/13
Age of walking alone	NA			−			−				3 years			1/13
No language expression	1 child was reported			+			+				+	+		12/13
Seizure	1 child did not have seizures			+			1 child				−	+		8/13
Hypotonia	−			+			Present before 1 year				+	−		5/13
Muscle spasm	NA			NA			After 1 year				−	−		4/13
Active patellar tendon reflex	NA			+			NA				−	−		3/13
Ophthalmologic features														
Strabismus	NA			NA			+				−	+		4/13
Nystagmus	NA			NA			+				−	−		4/13
Heterochromia iris	+			NA			NA				−	−		3/13
Optic atrophy	NA			NA			3 children				−	−		3/13
Abnormality of retinal pigmentation	NA			NA			1 child				−	−		1/13
Dysmorphic features														
Brachycephaly	−			−			−				+	−		1/13
Microcephaly	+			+			+				−	+		12/13
Hypertelorism	NA			+			NA				−	−		3/13
Micrognathia	NA			+			NA				−	−		3/13
Growth														
Short stature observed	+			NA			+				−	+		9/13
Term labor	NA			+			NA				+	+		6/13
Brain MRI	N.A			Hypoplastic corpus callosum in 2 children			Hypoplastic corpus callosum, abnormal myelination. 2 children with arachnoid cyst				Corpus callosum is slightly thine	NA		

*Note:* “+”: presence, “−”: not presence.

Abbreviations: Compound het, compound heterozygous; F, female; M, male; NA, not available.

Herein, we identified a novel variant in *EEF1D* through whole exome sequencing (WES). The aim of this study is to expand the phenotypic spectrum of NEDTCHAL associated with *EEF1D* variants. By identifying a novel variant in an Iranian sibling, we aim to further characterize the clinical manifestations of this disorder and contribute to a broader understanding of its phenotypic variability. This study not only provides additional evidence for the role of *EEF1D* in neurodevelopmental disorders but also helps to refine the phenotypic features associated with these conditions.

## 2 Materials and Methods

### 2.1 Patients Recruitment

Genetic counseling was offered to a consanguineous family with a 14‐year‐old girl experiencing seizures, neurodevelopmental delay, microcephaly, failure to thrive, cyanosis, and strabismus. Notably, her brother is also affected with similar symptoms. Written informed consent was obtained from the legal guardians for the publication of clinical data. The study was being performed in accordance with the ethical standards laid down in the 1964 Declaration of Helsinki and its later amendments (World Medical Association 2014).

### 2.2 Genetic Workup and Protein Analysis

WES was conducted to determine the molecular cause in this family. Genomic DNA was isolated using the salting out method. The Agilent SureSelect exome kit (Version 6) was used for DNA library preparation, DNA target enrichment, and capturing. The DNA libraries were sequenced using a Novaseq 6000 sequencer, achieving 100× coverage. The bioinformatic analysis followed the procedure outlined in our previous publication (Ashrafi et al. 2023; Tavasoli et al. 2022; Mohammadi et al. 2021; Ghoreyshi et al. 2025). In summary, the sequencing quality was verified using FastQC software. Subsequently, the raw reads were aligned to the GRCh38/hg38 reference genome using Burrows‐Wheeler Aligner (BWA) tool. Variant calling was performed using the Haplotype Caller tool of GATK toolkit package, and annotation was carried out using the VEP ensemble annotator. Variants with a minor allele frequency (MAF) greater than 1% were excluded by consulting various human population databases, including the Genome Aggregation Database (gnomAD), Exome Variant Server, 1000 Genomes, dbSNP151, and the Exome Aggregation Consortium version 0.3 database (ExAC). Sanger sequencing was used for confirmation, and the parents also underwent Sanger sequencing testing. To assess the potential impact of the variants on splicing, several prediction tools were utilized: HSF (Human Splicing Finder): This tool was used to predict the effect of the variant on splicing by evaluating its impact on splice site recognition and overall splicing efficiency. HSF integrates multiple algorithms to determine the likelihood of the variant affecting splicing. MaxEnt: This tool focuses on predicting changes in the strength of splice donor and acceptor sites. It was used to assess whether the deletion of the 2‐nucleotide splice acceptor site weakens the splice site and affects proper mRNA splicing. SpliceAI: SpliceAI was employed to predict whether the variant disrupts the normal splicing process by evaluating how it affects both donor and acceptor splice sites and the potential for creating novel splicing sites. Pangolin: This tool was used to analyze and predict the potential impact of genetic variants on RNA splicing, including evaluating the likelihood that this variant results in exon skipping or the introduction of cryptic splice sites. Pymol software: Pymol was utilized to visualize the potential structural effects of the variant on the protein produced by the gene. While not specifically for splicing, Pymol provides valuable insight into how changes in splicing could potentially affect the protein structure. UniProt: UniProt was used to gather functional information on the *EEF1D* and its protein products. It provided a comprehensive overview of known splicing events, protein isoforms, and functional domains that could be impacted by the variant.

## 3 Result

### 3.1 Clinical Features

The pedigree of the family with the two affected siblings with seizures, neurodevelopmental delay, microcephaly, failure to thrive, cyanosis and strabismus is shown in Figure 1A. The female sibling, currently 14 years old, was born at term to consanguineous parents after an uneventful pregnancy and normal vaginal delivery. Her birth weight was 3400 g (approximately 50th percentile for term newborns) and length was 50 cm (approximately 50th percentile). Within the first year of life, she developed microcephaly, with head circumference measured at 34 cm (< 3rd percentile for age, corresponding to severe microcephaly). At 4 months of age, she developed generalized tonic seizures, which recurred during infancy and early childhood. EEG later demonstrated epileptiform discharges, despite no active clinical seizures in recent years. She exhibited hypotonia, poor feeding, and suckling dysfunction from birth, requiring nasogastric feeding, which was later transitioned to gastrostomy. At 1 year of age, she was nonverbal, unable to sit, crawl, or walk, and showed no response to simple commands. She has remained wheelchair‐bound, with persistent axial hypotonia and global developmental delay. Ophthalmological findings included convergent strabismus and frequent blinking, with no structural ocular abnormalities. Electrodiagnostic studies were unremarkable. Metabolic screening, including plasma amino acids, urine organic acids, and acylcarnitine profiling, was within normal limits. No dysmorphic features or organomegaly were observed. Her current cognitive and motor status remain severely impaired.

The male sibling, now 7 years old, was born at 37 weeks of gestation via normal vaginal delivery. His birth weight and length were 2800 g (approximately 25th–50th percentile for 37 weeks of gestation) and 50 cm (approximately 50th percentile), respectively, similar to his sister. During infancy, he also developed progressive microcephaly, with a head circumference of 34 cm recorded within the first year of life (< 3rd percentile for age, consistent with severe microcephaly). He experienced febrile reactions following his 2‐month vaccination, characterized by intermittent fever and poor feeding. At 56 days of age, he developed perioral cyanosis, which prompted admission to the Neonatal Intensive Care Unit (NICU). Clinical evaluation revealed laryngomalacia, while chest X‐ray, abdominal ultrasound, and echocardiography were unremarkable except for mild peripheral pulmonary stenosis (PPS), small PDA, and mild tricuspid regurgitation (TR)—none of which were hemodynamically significant. Pulmonary evaluation ruled out other causes of cyanosis.

He exhibited neonatal‐onset seizures, and during early childhood, he was treated with a combination of Levetiracetam, Topiramate, and Carbamazepine. Seizures occurred about twice a year until age 4 and then gradually subsided. He is currently off antiepileptic medication. EEG remained normal throughout follow‐up, and nerve conduction studies were also normal. He presented with severe global developmental delay, hypotonia, and failure to thrive, with a weight of 7 kg at 19 months. At 1 year, he had no speech, no response to auditory stimuli, and was unable to sit, crawl, or walk. Currently, he remains non‐ambulatory and non‐verbal, with persistent strabismus, frequent blinking, and axial hypotonia. MRI revealed demyelination, white matter loss, and cerebellar atrophy. A comprehensive metabolic evaluation at 56 days of age, including GC/MS, LC–MS/MS, and acylcarnitine profile, was negative for more than 70 metabolic disorders.

### 3.2 Genetic Characteristics

In this family, a biallelic splice junction loss variant [chr8(hg38)‐143580722‐TACGTGCTGCCACAGGGGAAGGGACAGGAGGC>T; NM_001130053.5:c.1489‐26_1493del] was identified in *EEF1D* in 2 affected individuals through WES. This variant is located at the splice junction site of intron 7 of *EEF1D*. This variant was not found in gnomAD and Iranome databases. The carrier status of the parents was confirmed through Sanger sequencing (see Figure 1B). According to the ACMG/AMP guidelines, this variant was classified as likely pathogenic (PVS1, PP4 and PM2) and considered as the cause of disease in this family. *EEF1D* remains conserved during evolution through different organisms with a high degree of amino acid sequence similarity and identity between the orthologues (Figure 1C).

## 4 Discussion

The EEF1D protein is composed of two distinct functional domains that play critical roles in its activity: EF‐1 Beta Acid Domain (Positions 525–552), which this domain is essential for the protein's elongation factor function, and EF1B Domain (Positions 564–647), which this domain is equally crucial for the protein's overall functional integrity (Xu et al. 2022). Several disease‐causing variants in *EEF1D* have been identified and mapped along its sequence, with potential impacts on the protein's structure and function (Figure 1D).

*EEF1D* was first proposed as a candidate gene for ARID following its identification in a Syrian family with three children suffering from severe ID, microcephaly, and short stature. This discovery was a part of a larger study involving exome sequencing of 152 consanguineous families with neurodevelopmental disorders (Reuter et al. 2017). Further support for the gene's relevance came from the identification of a nonsense variant [(NM_001130053.5:c.948G>A; p.Trp316Ter)] in a Turkish family presenting with ID and developmental delays (Ugur Iseri et al. 2019). Both of these variants, reported by Reuter et al. (2017) and Ugur Iseri et al. (2019), specifically target the large exon present only in the transcripts for the eEF1BδL isoform (Kaitsuka et al. 2011). Notably, because these variants occur in regions specific to the long isoform, they are unlikely to affect the transcripts encoding the shorter eEF1Bδ isoforms.

The hypothesis that the selective absence of eEF1BδL could replicate the consequences of the two *EEF1D* variants has been thoroughly investigated by the researchers who initially identified this isoform. Their studies have demonstrated that the targeted suppression of eEF1BδL impacts cellular survival during heat stress (Kaitsuka et al. 2011). More recently, they have created a mouse model with a targeted deletion of the large *EEF1D* exon, effectively knocking out eEF1BδL. These mice appeared to develop normally until they underwent fear conditioning tests (Kaitsuka et al. 2018). Subsequent to these tests, the mice exhibited reduced brain weight, degeneration in the hippocampus and midbrain, thinner cortical layers, and in some cases, audiogenic seizures. These observations imply that eEF1BδL may play a critical role in brain function, particularly in response to external stimuli. In a later study, Averdunk et al. (2023) used WES to identify two distinct homozygous truncating variants in *EEF1D*, each found in separate consanguineous families with severe intellectual disability, microcephaly, spasticity, and failure to thrive. Additional features included optic atrophy, poor feeding, and recurrent aspiration pneumonia. The identified variants are c.1780T>C, p.(Trp594Arg) and c.1905+1G>A in families from Turkey and Oman respectively (Averdunk et al. 2023). These variants, like those previously reported, affect the long isoform of EEF1D (eEF1BδL), which functions as a transcription factor regulating heat shock element (HSE) target genes.

Structural comparison of the p.Trp594Arg variant was performed using PyMol software. In the wild‐type form, the tryptophan residue at position 594 (Trp594) interacts with leucine at position 592 (Leu592) and aspartate at position 590 (Asp590), with interaction distances of 2.0 and 2.3 Å, respectively. After substitution with arginine (Arg594), the interactions with Leu592 and Asp590 remain, but the distances shift slightly to 2.0 and 2.1 Å. For the c.1905+1G>A variant (Averdunk et al. 2023), prediction tools like HSF and MaxEnt identified a disruption of the wild‐type donor site and activation of an alternative donor site, likely impacting splicing (https://hsf.genomnis.com/). Splice AI and Pangolin predictions also suggest that this variant [chr8(hg38):143580011‐C‐T] significantly affects *EEF1D* splicing, with high confidence in the following effects: loss of the donor splice site at 1 bp (near the variant) and gain of a donor splice site at −3 bp (upstream of the variant). However, the reported variant in Averdunk et al.'s (2023) study is found in the C‐terminal GEF domain, indicating that disruptions in the protein translation process may be contributing to neurodevelopmental abnormalities. Variants affecting either the alternatively spliced exon of the long isoform or the conserved GEF domain have both been associated with severe neurodevelopmental phenotypes, particularly those involving microcephaly and spasticity (Averdunk et al. 2023).

*EEF1D* encodes the eEF1Bδ protein, which functions as a guanine nucleotide exchange factor (GEF). This protein facilitates the release of eEF1A from the ribosome during the elongation phase of protein synthesis. The longer isoform, eEF1BδL, contains an alternatively spliced exon encoding a nuclear localization signal (NLS), which allows it to translocate to the nucleus. There, eEF1BδL acts as a transcription factor regulating genes with heat shock elements (HSEs). Under heat stress, expression of the shorter eEF1Bδ isoforms decreases, while levels of the longer eEF1BδL isoform increase, suggesting its specific involvement in the cellular stress response (Kaitsuka et al. 2011). The study by Reuter and Ugur Iseri et al. identified two loss‐of‐function variants that impact the alternatively spliced exon of eEF1BδL. However, the third study by Averdunk et al. (2023) is the first to report homozygous variants in the highly conserved C‐terminal GEF domain of EEF1D, which are associated with a severe neurodevelopmental disorder. This highlights the critical role of the GEF domain in neurodevelopment (Averdunk et al. 2023; Ugur Iseri et al. 2019).

Additionally, a recent study by Zhang et al. (2024) reported a Chinese patient with global developmental delay and severe intellectual disability carrying compound heterozygous variants in *EEF1D* (c.1905+1G>A and c.676C>T) (Zhang et al. 2024). Notably, the splice site variant c.1905+1G>A, previously described in other studies, disrupts the GEF domain of the protein, while the nonsense variant c.676C>T (p.Gln226Ter) introduces a premature stop codon in the N‐terminal region of the long eEF1BδL isoform. This variant is reported for the first time and is predicted to impair the transcriptional regulatory functions of eEF1BδL, including its role in the heat shock response pathway. The clinical phenotype of the patient overlaps with previously reported cases, including hypotonia and severe developmental delay; however, brachycephaly and the ability to walk independently were unique features. This report not only expands the mutational spectrum of *EEF1D* but also provides further evidence for isoform‐specific pathogenicity.

In a comprehensive genetic study involving 215 individuals with intellectual disability (ID) from 118 Middle Eastern families, WES of 188 individuals led to the identification of a homozygous *EEF1D* variant (c.1905+1G>A) in one family (Al‐Kasbi et al. 2022). Interestingly, this same variant had also been reported previously in the study by Averdunk et al. (2023), supporting its pathogenic significance. The recurrence of the c.1905+1G>A variant in two unrelated Omani families, as reported in both Averdunk et al. (2023), raises the possibility of a founder effect in the Omani population. Further haplotype analysis would be required to confirm this hypothesis.

In an Iranian family, a novel homozygous variant (c.1489‐26_1493del) in *EEF1D* (NM_001130053.5) was identified through WES. This variant specifically affects the splice junction site of intron 7 which is predicted to cause significant changes in auxiliary splicing sequences, including a −5 alteration in ESE/ESS motif ratios. This variant also activates a cryptic acceptor site and is likely to affect splicing through the loss of the wild‐type acceptor site (HSF). In addition, a new donor splice site is predicted, further implicating potential splicing alterations. The mutation is strongly predicted based on SpliceAI and Pangolin predictions and to cause a loss of an acceptor splice site in the *EEF1D* with high confidence.

While the two variants reported by Reuter and Ugur Iseri et al. are loss‐of‐function variants that affect the alternatively spliced exon of eEF1BδL, the present study is the first to report homozygous variants at the splice junction of intron 7, associated with a frameshift deletion and a severe neurodevelopmental disorder.

All previously reported individuals, as well as the two affected siblings in this study, presented with microcephaly and developmental delay/intellectual disability (ID). The two Iranian patients also exhibited short stature and muscular hypotonia. While none of the individuals from this study or in the studies by Ugur Iser, Averdunk et al. (2023) were able to sit, stand, or walk independently, one individual in the Reuter et al. study achieved assisted walking by age 15. Clinical features such as optic atrophy, poor feeding, and recurrent aspiration pneumonia were exclusively reported by Averdunk et al. (Reuter et al. 2017; Averdunk et al. 2023; Ugur Iseri et al. 2019; Al‐Kasbi et al. 2022). In contrast, the present study identified additional unique features for the first time, including intermittent fever, laryngomalacia due to hypotonia, frequent blinking, and axial hypotonia.

Considering the clinical observations from our patients, the underlying disease mechanism remains speculative. To gain further insight, additional functional studies are needed to elucidate the effects of mutant *EEF1D* on both the long and short protein isoforms. It is noteworthy that several genes involved in protein translation (such as *YARS1*) have been shown to possess non‐canonical functions beyond their primary roles. Accordingly, potential alternative functions of *EEF1D* should also be explored as possible contributors to disease pathogenesis.

To assess the expression pattern of *EEF1D* in the human brain at the cellular level, we utilized the publicly available Deeply Integrated human Single‐Cell Omics (DISCO) database. As shown in Figure 2, *EEF1D* is widely expressed in most or all regions of the brain. *EEF1D* is expressed in a range of neurons and glial cells including astrocytes, oligodendrocytes, excitatory neurons, inhibitory neurons, and microglia. These data suggest that *EEF1D* could be important for central nervous system development. This statement is supported by the gene expression pattern of *EEF1D* orthologues in other species as this gene is highly conserved during evolution (Li, Zhang, et al. 2022).

**FIGURE 2:**
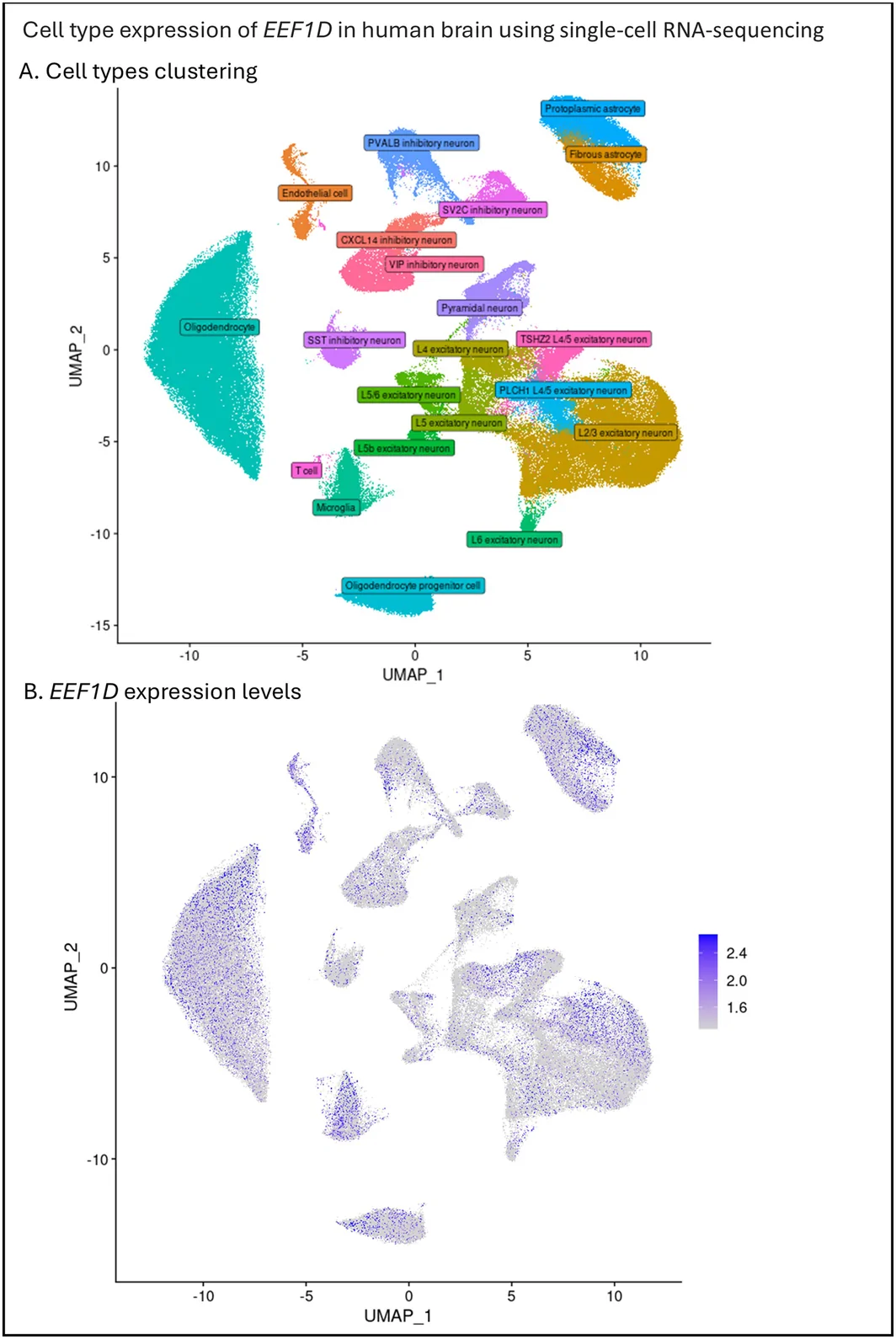
Cell type expression of *EEF1D* in human brain using a publicly available single‐cell RNA sequencing database, DISCO, shows that *EEF1D* is expressed in the brain. (A) Each cluster represents a specific cell type in the brain. (B) *EEF1D* is widely expressed in most of the cell types. Uniform Manifold Approximation and Projection (UMAP).

Single cell transcriptome sequencing in *C. elegans* shows that eef‐1B.1, orthologue of *EEF1D*, is widely expressed in neurons of the central nervous system (Ghaddar et al. 2023; Gao et al. 2024) (Figure 3A). Single cell RNA‐sequencing of *Drosophila Melanogaster* also shows that eEF1delta, ortholog of *EEF1D* in Drosophila, is broadly expressed in a set of neurons and glial cells in the central nervous system (Li, Janssens, et al. 2022) (Figure 3B). In addition, publicly available single‐cell sequencing of mouse shows that Eef1d, ortholog of *EEF1D* in mouse, indicates that this gene is broadly expressed throughout the body, including neuronal cells in the brain (Han et al. 2018; Wang et al. 2023) (Figure 3C).

**FIGURE 3:**
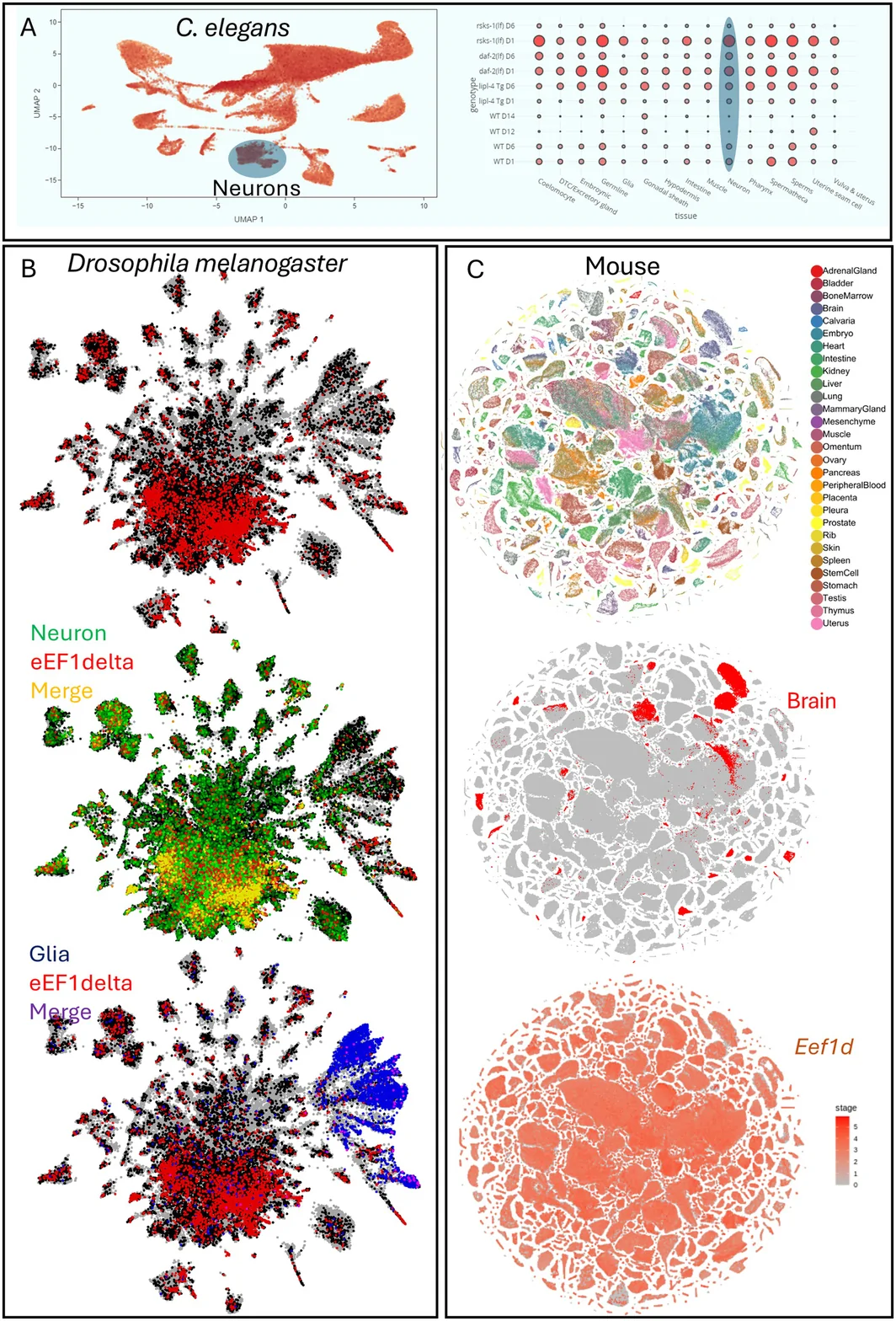
*EEF1D* is highly conserved throughout evolution across various organisms. (A) Single‐cell transcriptome sequencing in *C. elegans* reveals that eef‐1B.1, the ortholog of *EEF1D*, is widely expressed in neurons. Notably, downregulation of eef‐1B.1 through RNA interference (RNAi) leads to embryonic lethality, suggesting that this gene is crucial for the development of *C. elegans* . (B) Similarly, single‐cell RNA sequencing in *Drosophila melanogaster* shows that eEF1delta, the Drosophila ortholog of *EEF1D*, is broadly expressed in various neurons and glial cells within the central nervous system. (C) Furthermore, single‐cell sequencing data from mice indicates that Eef1d, the mouse ortholog, is also widely expressed throughout the body, including in neuronal cells of the brain. These findings collectively underscore the fundamental role of *EEF1D* in the development and function of the nervous system across different species.

Our clinical observation in human subjects and expression of *EEFD* in the human brain can support the importance of this gene in the development of CNS. In addition to human, the investigation of the orthologues in other species shows their essential role in neuronal development or function. In *C. elegans* , we observed that the gene is expressed in the CNS. Interestingly, downregulation of eef‐1B.1 using RNAi causes embryonic lethality. These interesting findings suggest that *eef‐1B.1* is crucial for the development of *C. elegans* (Kamath et al. 2003).

In mouse model, in line with the mouse single cell gene expression, gene expression data from Allen Institute shows that *Eef1d*, ortholog of *EEF1D* in mouse, is mainly expressed in cerebellum, hippocampus, and midbrain of the adult mouse suggesting that this gene could be important for motor coordination, cognition, learning and development of the nervous system (Daigle et al. 2018). In addition, loss of function allele generated by the International Mouse Phenotyping Consortium (IMPC), *Eef1d* ^*em1(IMPC)Mbp*^ allele, has been shown to have significant effects on embryonic development. When present in a homozygous state, this allele leads to severe deficits in embryonic development, culminating in preweaning lethality. This data suggests that *Eef1d* plays a critical role in early developmental processes necessary for the survival of the embryo. Moreover, heterozygous mutation in the *Eef1d* is associated with a range of multisystem developmental conditions. These findings indicate that even a single copy of the mutated allele can disrupt normal development, leading to various phenotypic manifestations in mouse. The phenotypic variability observed in heterozygous individuals underscores the importance of *Eef1d* in the proper development and viability of mice, highlighting its potential role as a key regulator in multiple biological systems. Overall, these findings emphasize the essential function of *Eef1d* in CNS development (Groza et al. 2023).

In summary, this study presents the first report of an *EEF1D*‐associated neurodevelopmental disorder in the Iranian population, adding new information to the existing knowledge about the genetic and clinical spectrum of this disorder. Together with previous studies, our findings show that disease‐causing variants in *EEF1D* can be associated with a diverse range of neurodevelopmental presentations, including autosomal recessive intellectual disability. These findings also suggest that WES can be a valuable tool for affected individuals with a possible diagnosis of genetic neurodevelopmental disorders. However, further functional studies are necessary to clarify how *EEF1D* dysfunction affects brain development and contributes to the associated clinical manifestations.

## Author Contributions

**Arastoo Kaki:** data curation, writing – review and editing, writing – original draft, conceptualization, methodology, validation, software. **Afshin Fayazi:** conceptualization, writing – original draft, writing – review and editing. **Behzad Rezaei:** conceptualization, writing – original draft, writing – review and editing. **Masoud Garshasbi:** supervision, conceptualization, data curation, writing – original draft, writing – review and editing, investigation, methodology, validation, formal analysis. **Pouria Mohammadi:** conceptualization, methodology, validation, visualization, writing – review and editing, writing – original draft, investigation, software, data curation, formal analysis, supervision. **Seyed Mohammad Kahani:** conceptualization, writing – original draft, writing – review and editing. **Maedeh Ganji:** writing – original draft, writing – review and editing, investigation, conceptualization, methodology, validation, visualization, software, formal analysis, project administration, data curation, supervision. **Ali H. Bereshneh:** conceptualization, investigation, writing – original draft, writing – review and editing, methodology, data curation. **Mohammad Farid Mohammadi:** conceptualization, writing – original draft, writing – review and editing, methodology, data curation, software, formal analysis, visualization, investigation. **Javad Behroozi:** conceptualization, methodology, writing – review and editing, writing – original draft, data curation.

## Funding

The authors have nothing to report.

## Ethics Statement

The study was performed in accordance with the ethical standards laid down in the 1964 Declaration of Helsinki and its later amendments.

## Consent

Written consent was obtained from parents as the legal guardians of the proband.

## Conflicts of Interest

The authors declare no conflicts of interest.

## Data Availability

We state that the data not provided in this article will be shared on request by email to the corresponding author from any qualified investigator for purposes of replicating procedures and results.
